# Highly Anisotropic GeSe Nanosheets for Phototransistors with Ultrahigh Photoresponsivity

**DOI:** 10.1002/advs.201800478

**Published:** 2018-06-21

**Authors:** Xing Zhou, Xiaozong Hu, Bao Jin, Jing Yu, Kailang Liu, Huiqiao Li, Tianyou Zhai

**Affiliations:** ^1^ State Key Laboratory of Material Processing and Die & Mould Technology School of Materials Science and Engineering Huazhong University of Science and Technology (HUST) Wuhan 430074 P. R. China

**Keywords:** 2D GeSe, anisotropy, phototransistors

## Abstract

2D GeSe possesses black phosphorous‐analog‐layered structure and shows excellent environmental stability, as well as highly anisotropic in‐plane properties. Additionally, its high absorption efficiency in the visible range and high charge carrier mobility render it promising for applications in optoelectronics. However, most reported GeSe‐based photodetectors show frustrating performance especially in photoresponsivity. Herein, a 2D GeSe‐based phototransistor with an ultrahigh photoresponsivity is demonstrated. Its optimized photoresponsivity can be up to ≈1.6 × 10^5^ A W^−1^. This high responsivity can be attributed to the highly efficient light absorption and the enhanced photoconductive gain due to the existence of trap states. The exfoliated GeSe nanosheet is confirmed to be along the [001] (armchair direction) and [010] (zigzag direction) using transmission electron microscopy and anisotropic Raman characterizations. The angle‐dependent electric and photoresponsive performance is systematically explored. Notably, the GeSe‐based phototransistor shows strong polarization‐dependent photoresponse with a peak/valley ratio of 1.3. Furthermore, the charge carrier mobility along the armchair direction is measured to be 1.85 times larger than that along the zigzag direction.

## 1 Introduction

Black phosphorous (BP) has attracted significant attention due to its remarkable electrical, optical, and optoelectronic properties including tunable direct band gap^1^, ^2^, ^3^ and high carrier mobility.^4^, ^5^, ^6^ Specifically, the sp^3^ nonequivalent hybridization between adjacent P atoms results in the puckered structure, leading to in‐plane anisotropic characters such as anisotropic mechanical, electrical, and optical properties.^7^, ^8^, ^9^ These anisotropic properties provide a new degree of freedom for modulating its electrical and optical properties, which may supply a new platform for multifunctional electronics and optoelectronics.^10^ However, BP is extremely unstable in ambient environment due to the photo‐oxidation effect,^11^, ^12^ which impedes its further applications. Thus exploring other 2D materials with air stability and novel functionalities is desperately required.

As an important member of 2D group IV‐VIA semiconductors, GeSe is a layered material of BP‐analog structure, in which three Se atoms are coordinated to one Ge atom to form the puckered Ge‐Se layers. 2D GeSe shows excellent air stability and highly in‐plane anisotropic properties due to the low symmetry of *Pnma* space group.^13^ GeSe is a p‐type semiconductor with closely located direct and indirect band gaps in the range of 1.1–1.2 eV,^13^, ^14^ which matches well with solar spectrum.^15^ Besides, GeSe exhibits a high absorption coefficient of ≈10^5^ cm^−1^ 16 in the visible range and a high hole mobility of 128.6 cm^2^ V^−1^ s^−1^,^17^ rendering it promising candidate for electronics and optoelectronics.^18^, ^19^, ^20^ What's more, the low‐symmetry structure may arouse unprecedented physical properties such as anisotropy providing a new degree of freedom for regulating the optical and electronic properties.^21^ Recently, GeSe nanosheet‐based photodetectors have been constructed and showed great potential applications. However, these reported photodetectors based on GeSe nanosheets exhibited poor responsivity such as ≈3.5 A W^−1^ 22 and 4.25 A W^−1^.^13^ What's more, the in‐depth investigations of anisotropic charge carrier transportation and photoresponsive process are still lacking.

In this work, a 2D GeSe‐based phototransistor with an ultrahigh photoresponsivity is demonstrated. The optimized photoresponsivity can reach 1.6 × 10^5^ A W^−1^ (under illumination of 532 nm light), which is about five orders of magnitude higher than those of previously reported GeSe‐based photodetectors^13^, ^22^ and 100 times higher than that of monolayer MoS_2_.^23^ As far as we know, this is better than most reported responsivities of similarly structured phototransistors based on single pristine 2D materials^13^, ^22^, ^23^, ^24^, ^25^, ^26^, ^27^, ^28^ without further hybridization and functionalization. The high responsivity can be attributed to the highly efficient light absorption as well as the enhanced photoconductive gain resulting from the existence of trap states. Then we systematically explored the in‐plane properties including structural, vibrational, electrical, and photoresponsive anisotropies. Assisted by transmission electron microscopy (TEM) and polarization‐resolved Raman characterizations, the cleavage directions of the exfoliated GeSe nanosheet are confirmed to be along the [001] (armchair direction) and [010] (zigzag direction). This GeSe‐based phototransistor also shows strong polarization‐dependent photoresponse with a peak/valley ratio of 1.3. Furthermore, the mobility along armchair direction is measured to be 1.85 times larger than that along the zigzag direction. Our results point to 2D GeSe to be a promising candidate for future optoelectronic applications.

## 2 Results and Discussion

**Figure 1:**
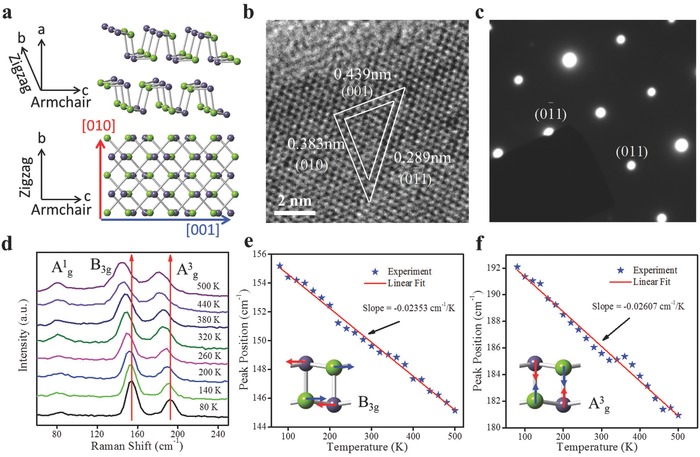
a) Crystal structure of GeSe. b) HRTEM image of 2D GeSe nanosheet. c) SAED pattern of GeSe. d) Temperature‐dependent Raman spectra from 80 to 500 K under 532 nm wavelength. e,f) Temperature‐dependent phonon peaks of B_3g_ and A^3^_g_ modes.

GeSe possesses a puckered structure similar to BP as demonstrated by the atomic model in **Figure** **1**a. Commonly, the *c*‐axis is named as the armchair direction, and the *b*‐axis is designated as the zigzag direction.^13^ The 2D GeSe crystals were exfoliated from bulk GeSe. TEM was employed to explore the crystal structure of 2D GeSe. As demonstrated in Figure 1b, a high‐resolution TEM (HRTEM) image confirms the high crystalline quality of the exfoliated nanosheet. The lattice fringes of (001) and (010) planes are apparent and measured to be 0.439 and 0.383 nm, respectively.^18^, ^19^ Figure 1c shows the selected area electron diffraction (SAED) pattern with sharp diffraction spots, matching well with that of the bulk GeSe.

Then Raman spectroscopy was performed to further investigate the crystal structure of the exfoliated 2D GeSe nanosheets. Figure 1d illustrates the temperature‐dependent Raman spectra measured at the temperature range from 80 to 500 K, which is a practical way for uncovering the atomic bonds, thermal expansion, and phonon vibration properties of 2D materials.^21^, ^29^, ^30^, ^31^, ^32^ The peak positions around 82.5, 149.2, and 185.9 are corresponding to the A^1^_g_, B_3g_, and A^3^_g_ modes, respectively, in good agreement with the previous reports.^13^, ^33^ As demonstrated in Figure 1d, all these visible peaks exhibited redshift and softening with increasing temperature due to the anharmonic electron–phonon coupling, phonon–phonon interactions, or thermal expansion.^32^ For example, both B_3g_ and A^3^_g_ peak positions showed linear redshifts by about 10 and 11 cm^−1^ with increasing temperature from 80 to 500 K (Figure 1e,f). The temperature‐dependent peak positions can be fitted by a linear equation with a first‐order temperature coefficient (χ): ω(*T*) = ω_0_ + *χT*, where ω_0_ is the peak position of B_3g_ and A^3^_g_ modes at 0 K, *T* is the temperature, and χ is the first‐order temperature coefficient correlated with temperature‐dependent peak shift of nanomaterials. Accordingly, the fitted values of χ for B_3g_ and A^3^_g_ modes are −0.02353 and −0.02607 cm^−1^ K^−1^, respectively. The variation of phonon frequency with temperature may result from the anharmonicity between different atoms at constant pressure including the contraction of the crystal and the thermal expansion. The variation of phonon frequency can be determined by^29^

(1) $$\begin{array}{l} \;\;\;\;\;\;\;\;\begin{array}{c} \Delta \omega =(\chi_{T}+\chi_{V})\Delta T={\left(\frac{d\omega }{dT}\right)}_{V}\Delta T+{\left(\frac{d\omega }{dV}\right)}_{T}\Delta V\\ \begin{array}{l} \;={\left(\frac{d\omega }{dT}\right)}_{V}\Delta T+{\left(\frac{d\omega }{dV}\right)}_{T}{\left(\frac{d\omega }{dT}\right)}_{P}\Delta T \end{array} \end{array} \end{array}$$

where χ = χ_*T*_ + χ_*V*_, in which *χ_T_* is the shift of self‐energy induced by the phonon modes coupling and *χ_V_* is the shift resulting from volume change caused by thermal expansion.^29^, ^30^ Notably, the calculated first‐order temperature coefficients of GeSe are larger than those of MoS_2_ (A_1g_ mode: −0.011 cm^−1^ K^−1^)^34^ and SnSe_2_ (A_1g_ mode: −0.0129 cm^−1^ K^−1^),^30^ while comparable to those of BP (A_2g_ mode: −0.0283 cm^−1^ K^−1^)^35^ and SnS flake (A_g_ mode: −0.023 cm^−1^ K^−1^).^36^ It has been proposed that the first‐order temperature coefficients of 2D materials are related to the van der Waals interaction between the adjacent layers.^29^ For SnSe_2_ and MoS_2_, the weak interaction between adjacent layers may induce the smaller first‐order temperature coefficients. The higher coefficients of GeSe may result from the unique puckered crystal structure, which is similar to those of BP and SnS.^35^, ^36^

**Figure 2:**
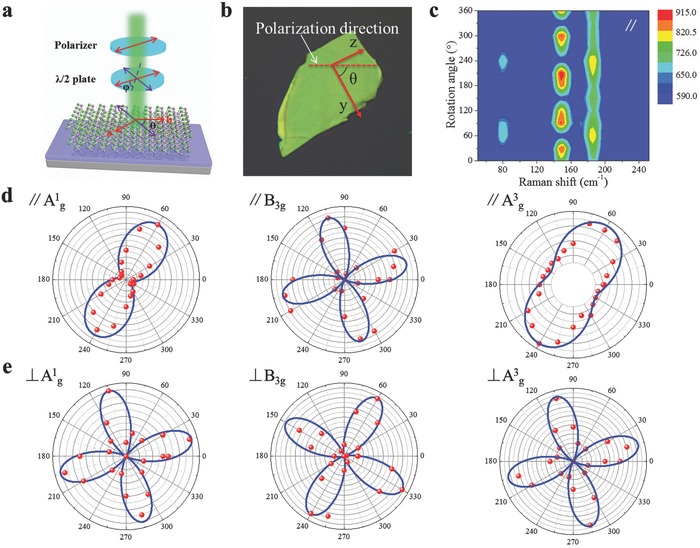
Polarized Raman spectra of GeSe nanosheet. a) Illustration of the angle‐resolved Raman spectra measurements. b) Optical image of the measured nanosheet. c) False‐color plot of the polarized Raman spectra under parallel configuration, where the color scale indicates the intensity of the Raman vibration. d) Angle‐resolved Raman scattering intensities of A^1^_g_, B_3g_, and A^3^_g_ modes under parallel configuration. e) Angle‐resolved Raman scattering intensities of A^1^_g_, B_3g_, and A^3^_g_ modes under cross configuration.

**Figure** **2**a shows the illustration of the angle‐resolved polarized Raman spectroscopy measurements. The optical image of the exfoliated 2D GeSe is demonstrated in Figure 2b. Herein, we defined the *c*‐axis (armchair direction) as the *z*‐axis, and the *b*‐axis (zigzag direction) as the *y*‐axis based on the crystal structure and TEM characterizations. The incident light was polarized along the horizontal direction and the analyzer was inserted at the entrance of the spectroscope so that we can analyze the Raman scattering signals from parallel or cross‐polarization configurations, respectively. The angle‐dependent Raman spectra were realized by revolving the sample in the *y–z* plane and the angle θ (Figure 2b) was defined as the angle between the light polarization direction and the zigzag direction (*b*‐axis).

A series of angle‐dependent Raman spectra were performed under parallel (Figure S1a, Supporting Information) and cross (Figure S1b, Supporting Information) configuration. It is obvious that all these three phonon peak intensities of A^1^_g_, B_3g_, and A^3^_g_ modes exhibit strong angle dependence as demonstrated by the contour color map under parallel‐polarized configuration (Figure 2c). Since the A^1^_g_ and A^3^_g_ modes both belong to Raman active mode A_g_, the peak intensities of A^1^_g_ and A^3^_g_ modes show the same periodic variation with changing rotation angle under parallel and cross‐polarized configuration, while B_3g_ mode shows different variation rule. To further explore the angle‐dependent Raman scattering intensity, we summarized these three peak intensities with different rotation angle under parallel and cross‐polarized configuration in Figure 2d,e. The anisotropic Raman spectra of GeSe can be well understood according to the classical Placzek model, in which the Raman scattering intensity can be deduced as^13^, ^37^ $I\propto \;\;|e_{i}\cdot R\cdot e_{s}{|}^{2}$, where *e*_i_ and *e*_s_ are the unit polarization vectors of the incident and scattered light, respectively, and *R* is the Raman tensor for the Raman active modes of GeSe. The incident light polarization *e*_i_ = (0, cosθ, sinθ), while *e*_s_ = (0, cosθ, sinθ)^T^ under parallel‐polarized configuration and (0, −sinθ, cosθ)^T^ under cross‐polarized configuration.^38^, ^39^, ^40^ For the orthorhombic crystal structure belonging to the D_2h_ space group, the Raman tensors of B_3g_ and Ag modes can be described as below^13^

(2) $$\begin{array}{c} R(A_{g})=\left(\begin{array}{ccc} A & 0 & 0\\ 0 & B & 0\\ 0 & 0 & C \end{array}\right),\;\;\;\;\;R(B_{1g})=\left(\begin{array}{ccc} 0 & D & 0\\ D & 0 & 0\\ 0 & 0 & 0 \end{array}\right)\\ R(B_{2g})=\left(\begin{array}{ccc} 0 & 0 & E\\ 0 & 0 & 0\\ E & 0 & 0 \end{array}\right),\;\;\;\;\;\;R(B_{3g})=\left(\begin{array}{ccc} 0 & 0 & 0\\ 0 & 0 & F\\ 0 & F & 0 \end{array}\right) \end{array}$$

Herein, only A_g_ and B_3g_ modes can be detected due to the nonzero values in the Raman tensors. Thus the Raman scattering intensities of A_g_ and B_3g_ modes can be further expressed by^13^, ^37^

(3) $$I(A_{g},//)={\left(B\cos^{2}\theta +C\sin^{2}\theta \right)}^{2}$$

(4) $$I(B_{3g},//)=F^{2}\sin^{2}2\theta$$

(5) $$I(A_{g},⊥)=\frac{{\left(C-B\right)}^{2}}{4}\sin^{2}2\theta$$

(6) $$I(B_{3g},⊥)=F^{2}\cos^{2}2\theta$$

Thus the angle‐dependent Raman scattering intensities were well fitted with Equations (3)–(6). It is apparently observed that A^1^_g_ and A^3^_g_ modes show a marked periodic variation feature of 180° under parallel‐polarized configuration, while B_3g_ mode shows a periodic variation feature of 90°. However, all of these three modes show a periodic variation feature of 90° under cross‐polarized configuration. The intensities calculated from the abovementioned equations have always reached the maximum at θ = 0° and 90°. Thus the crystalline orientation of the exfoliated GeSe nanosheet can be determined by the A_g_ mode.

**Figure 3:**
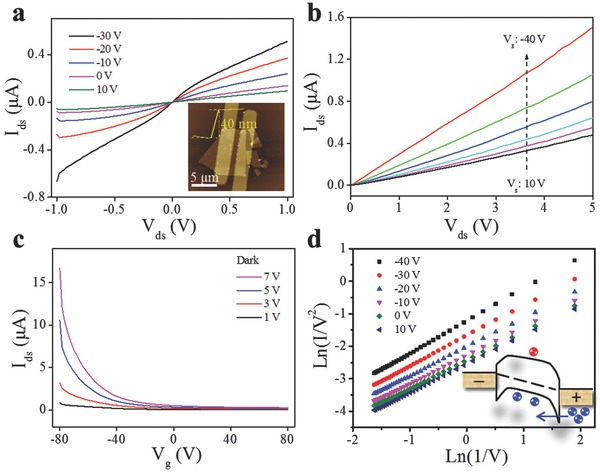
a) *I*_ds_–*V*_ds_ curves at different gate voltages. Inset: atomic force microscopy (AFM) image of the device. b) Output curves at different gate voltages. c) Transfer characteristics at different drain–source voltages. d) Direct tunneling plots at different gate voltages. Inset: Illustration of carrier tunneling.

To probe the electrical properties, we fabricated a GeSe nanosheet (≈40 nm, in the inset of **Figure** **3**a) based phototransistor via a back‐gated field effect transistor (FET) construction. The *I*_ds_–*V*_ds_ characteristics at different gate voltages are shown in Figure 3a and the output characteristics are demonstrated in Figure 3b. The linearity of *I*_ds_–*V*_ds_ curves indicates the ohmic contact between the electrodes and GeSe nanosheet. Figure 3c illustrates the transfer characteristics exhibiting typical p‐type semiconducting behavior. The hole mobility (*µ*) of the device can be calculated by $\mu =\frac{dI_{\mathrm{ds}}}{dV_{\mathrm{ds}}}\cdot \frac{L}{WC_{i}V_{\mathrm{ds}}}$, where *W* and *L* are the width and length of the device, and *C*_i_ is the capacitance per unit area (11.6 nF cm^−2^ for 300 nm SiO_2_ layer in these experiments). The calculated on/off ratio and *µ* are ≈10^3^ and 0.8 cm^2^ V^−1^ s^−1^ at room temperature with *V*_ds_ = 1 V, which are comparable with those of reported MoS_2_,^41^ SnSe_2_,^28^ and SnS_2_ 25, 42 FETs recently. To further explore the charge transport mechanism, we employed the carrier injection models such as thermionic emission and tunneling.^43^, ^44^, ^45^ Commonly, thermionic emission (Schottky emission), direct tunneling, and Fowler–Nordheim tunneling mainly dominate charge transport at a metal–semiconductor contact.^43^ Thus the *I*_ds_–*V*_ds_ curves can be fitted with a direct tunneling process with Simmons approximation with gate voltages varying from −40 to 10 V as illustrated in Figure 3d: $I_{\mathrm{DT}}\propto V\exp\left(-\frac{4\pi d\sqrt{2m^{*}\phi }}{h}\right)$, where *h*, m*, *d*, and φ are Plank constant, effective electron mass, tunneling thickness, and tunneling barrier, respectively. The linearity of the well‐fitted curves of Ln(*I*/*V* ^2^) versus Ln(*V* ^−1^) suggested that carriers can directly tunnel through the barrier due to the small barrier at the metal–semiconductor interface as shown in the inset of Figure 3d.

**Figure 4:**
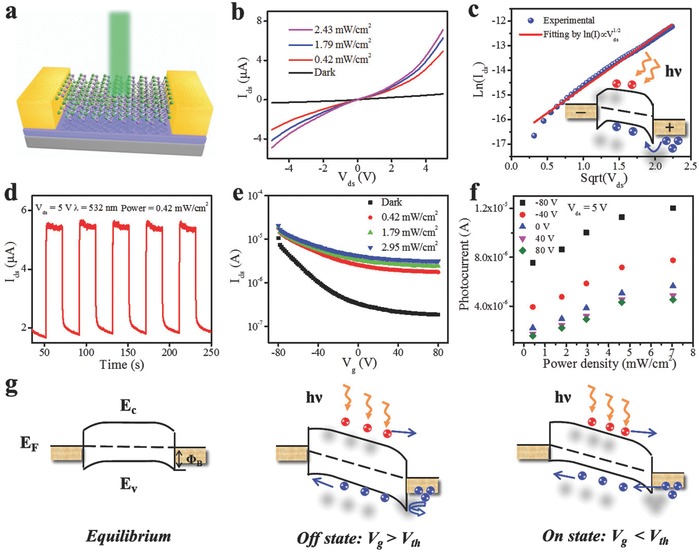
a) Schematic of the photodetector. b) *I*_ds_–*V*_ds_ curves in the dark and under light illumination with different power intensities. c) Thermionic emission plot. Inset: Illustration of carrier transport. d) Time‐resolved photoresponse at *V*_ds_ = 1 V. e) Transfer curves in the dark and under light illumination with different power intensities. f) Power dependence of photocurrent. g) Energy band diagrams of GeSe‐based phototransistor taking into consideration the small Schottky barriers at the electrodes/channel interface.

Furthermore, we probed the photoresponse of this GeSe‐based phototransistor (**Figure** **4**a). Figure 4b shows the photoresponse curves at different illuminated power intensities of the incident light (532 nm). Notably, the curves showed deviation from the linear relationship under illumination, which were different from the case in the dark. We further found that the *I*_ds_–*V*_ds_ curves can be well‐fitted by the Schottky emission model under illumination: Ln(*I*) versus *V* ^1/2^ plot showing linear dependence (Figure 4c).^44^, ^46^ This result indicated that the large amounts of photoinduced carriers reduce the barrier height at the metal–semiconductor interface and the Schottky emission mechanism comes to dominate the carrier transport (inset of Figure 4c). The cycling characteristics of the phototransistor were also probed under illumination of 532 nm with the power intensity of 0.42 mW cm^−2^. As demonstrated in Figure 4d, the constructed GeSe‐based phototransistor exhibits excellent stability during the measurement. The enlarged view of one response cycle shows that the response and decay rates are ≈0.28 and 0.51 s (Figure S2, Supporting Information), respectively. Besides, it is obvious that the decay process composed of two parts. The first part is the fast decay process, in which the photocurrent dropped quickly in 4 ms when the illumination is turned off, and the second part is the subsequent slow decay process lasting for several hundreds of milliseconds. This slow decay process may result from the defects, impurities at the interface between SiO_2_ substrate and GeSe surface, or the interior trap states.^47^ To further determine the photoresponse performance, the responsivity *R_λ_* was calculated to be ≈8.4 × 10^4^ A W^−1^ by the equation: *Rλ* = *I*_ph_/*PS*,^48^, ^49^ where *I*_ph_ = *I*_light_ – *I*_dark_ (*V*_ds_ = 5 V, *V*_g_ = 0 V), *P* is the light intensity (0.42 mW cm^−2^), and *S* is the effective area under illumination (≈8 µm^2^). This result is four orders of magnitude higher than those of GeSe‐based photodetectors reported by others,^13^, ^22^ and can rival or even surpass many other reported 2D material based photodetectors.^50^, ^51^ Then the obtained external quantum efficiency (EQE) was calculated to be ≈1.9 × 10^7^% via the formula: EQE = *hcR*_λ_/*eλ*, where *c* is the speed of light, λ is the incident light wavelength, and *e* is the unit electronic charge. Besides, detectivity (D*) represents the ability of a photodetector to determine weak optical signals, and can be expressed by D* = Δf · S^1/2^/*NEP*, in which *Δf* is the electrical bandwidth of the device and NEP is the noise equivalent power reflecting the minimum signal intensity for a photodetector detecting a signal from noise; whereas, if the NEP value is low, D* can be further predigested as D* = *R*_λ_ *S* ^1/2^/(2*eI*_dark_)^1/2^ and was estimated to be ≈3.7 × 10^13^ Jones. The gate‐bias‐dependent photoresponse was further explored. Figure 4e demonstrates the transfer characteristics of the phototransistor in the dark and under illumination with different light intensities (532 nm). It is observed that photocurrent rises at the whole range of gate bias, suggesting that the photocurrents dominate over tunneling and thermionic currents with the operation by gate bias.^52^, ^53^ The gate‐dependent photoresponse can be explained by the energy band diagrams in Figure 4g. The device is characterized by the direct tunneling process at the equilibrium state (*V*_g_ = 0 V, without light illumination) as demonstrated above. The photocurrent rises linearly with *V*_ds_ due to the decrease of the carrier transit time when the device is at OFF state (*V*_g_ > *V*_th_ = –20 V). Thus the dark current can be effectively suppressed by the higher barrier at OFF state resulted in more sensitive at *V*_g_ > *V*_th_. Whereas, tunneling and thermionic currents also contribute to the collected currents due to lower barrier at the metal–semiconductor interface at ON state (*V*_g_ < *V*_th_), leading to rising photoresponse.^23^ Thus the responsivity of this phototransistor can be further improved to 1.6 × 10^5^ A W^−1^ at *V*_g_ = −80 V (EQE = 3.9 × 10^7^%, D* = 2.9 × 10^13^ Jones). As far as we know, this is better than most reported responsivities of similarly structured phototransistors based on single pristine 2D materials^28^, ^54^ without further hybridization and functionalization. The comparison of photodetectors based on different 2D materials is summarized in **Table** **1**.^13^, ^22^, ^23^, ^24^, ^25^, ^26^, ^27^, ^28^ The intrinsic responsivity *R*_0_ and the photoconductive gain *G* both contribute to the total responsivity:^55^ $R_{\lambda }\;=\;\;R_{0}\cdot G\;=\;\;\frac{\eta e}{hv}\cdot G$, where η is the quantum efficiency and υ is the frequency of incident light. The ultrahigh responsivity results from the enhancement of both the η and *G*. More specifically, the quantum efficiency η is proportional to the total amount of the absorbed photons *P*_d_, which is directly related to the thickness of 2D materials and can be expressed by *P_d_* = *P*_0_(1 − *e* ^−*αd*^), in which *P*_0_ is the total number of incident photons, α is the absorption coefficient, and *d* is the thickness of 2D materials. Herein, *P_d_* along with η can be notably increased due to large thickness of the multilayer GeSe nanosheet. The other main contribution for the ultrahigh responsivity may be the existence of trap states resulting in large *G*,^22^, ^55^ and the trap states mainly come from the Ge or Se vacancies, or the defects at the interface of GeSe and SiO_2_ substrate.^22^, ^47^ To further probe the trap states in GeSe, we extracted incident light power‐dependent photocurrent characteristics at different bias voltages in Figure 4f. Fitting the plots by power law *I*_ph_ ∝ *P* ^θ^, the factor θ can be extracted to be 0.38 at *V*_g_ = 0 V, and varies from 0.18 to 0.44 as *V*_g_ increases from −80 to 80 V. The severe deviation of linear relation between the photocurrent and the incident light power may be related to a joint effect of the Ge or Se vacancies in GeSe and the defects at GeSe/SiO_2_ interface due to the large surface aspect ratio. This sublinear dependence can also explain the photocurrent decay process, which can be apparently divided into a fast part and a slow part. Subsequently, the photogenerated carriers can fill some of the trap states in the band gap of GeSe, which will prolong the lifetime of excited carriers *T*_lifetime_ resulting in the significantly improved gain: *G* = *T*_lifetime_/*T*_transit_.

**Table 1:** Comparison of photodetectors based on different 2D materials. 1L: one layer; ML: multilayer

Device	*V*_g_ [V]	*R_λ_* [A W^−1^]	EQE [%]	D* [Jones]	Ref.
1L MoS_2_	−70	8.8 × 10^2^	–	–	23
ML MoS_2_	−3	1.2 × 10^−2^	–	–	24
ML GaTe	−30	8 × 10^2^	–	–	26
ML WS_2_	–	12.4	2.4 × 10^3^	9.2 × 10^11^	27
ML SnSe_2_	–	1.1 × 10^3^	2.6 × 10^5^	1.0 × 10^10^	28
ML SnS_2_	20	4 × 10^2^	–	3.0 × 10^10^	25
ML GeSe	–	3.5	5.3 × 10^2^	–	22
ML GeSe	–	4.2	–	–	13
ML GeSe	–80	1.6 × 10^5^	3.9 × 10^7^	2.9 × 10^13^	This work

**Figure 5:**
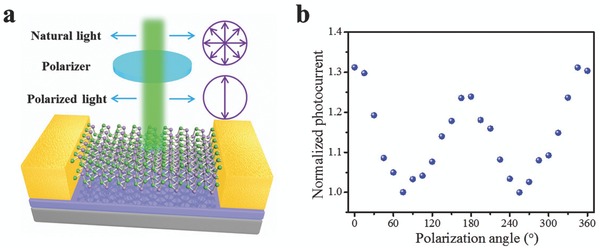
a) Schematic structure of GeSe‐based photodetector. b) Normalized photocurrent of GeSe‐based photodetector as a function of the polarization angle θ.

Since the Raman spectra of GeSe exhibited a high in‐plane anisotropic feature, it holds promising potential in the applications of linear linear‐polarization‐sensitive photodetectors.^56^ As illustrated in **Figure** **5**a, an analyzer was employed to vary the θ between the *c*‐axis direction of the material and the incident polarization direction. The photocurrent demonstrated three peaks at θ = 0°, 180°, and 360° with the light polarization parallel to the *c*‐axis direction of GeSe, while the valleys are at θ = 90° and 270° with the light polarization being perpendicular to the *c*‐axis direction of GeSe (Figure 5b). The peak to valley ratio is about 1.3, indicating the strong polarization‐sensitive photodetection.

**Figure 6:**
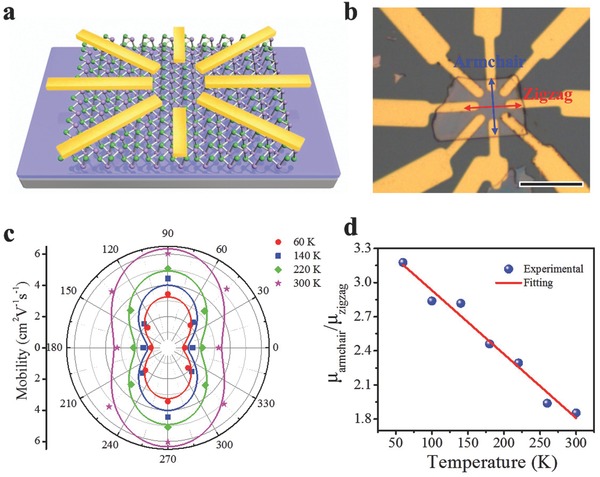
a) Schematic view of device structure. b) Optical image of the device with eight electrodes on GeSe. Scale bar: 10 µm. c) Angle‐dependent mobility at different temperatures from 60 to 300 K. d) Temperature‐dependent ratio of mobility along the armchair and zigzag directions.

Then the investigation of its electrical anisotropy was further performed based on multiterminal FET device illustrated in **Figure** **6**a. As shown in Figure 6b, eight electrodes were placed at an angle of 45°. Before electrical test, we employed angle‐resolved Raman spectra to determine the armchair and zigzag direction of the device. The incident laser polarization is along the horizontal indicating that θ starts from 0°. As demonstrated in Figure S3 (Supporting Information), it is observed that the Raman scattering intensities of A^1^_g_ and A^3^_g_ modes show a variation cycle of 180° and reach valley values at almost 0° and 180°, suggesting that the armchair direction is perpendicular to the horizontal direction in Figure 6b. Figure S4 (Supporting Information) demonstrates the temperature‐dependent‐transfer characteristics with different angles (*θ =* 0°, 45°, 90°, 135°). Casting a glance at these transfer curves, it is observed that the armchair direction exhibits higher mobility than that of the zigzag direction, which is similar with the reported BP recently.^57^ To further quantify these results, angle‐resolved hole mobilities at different temperatures have been plotted in Figure 6c. Obviously, the mobility along the armchair direction (≈6.03 cm^2^ V^−1^ s^−1^) is higher than that along the zigzag direction (≈3.25 cm^2^ V^−1^ s^−1^). Since μ∝*m* ^−1^ (*m* is the effective mass), the anisotropic electrical transport can be explained by the different effective masses along different directions.^38^ Theoretical calculations have demonstrated that the effective masses of GeSe are *m*_armchair_ = 0.16*m*_0_ and *m*_zigzag_ = 0.33*m*_0_ (*m*_0_ is the free‐electron mass).^58^ These theoretical calculations suggest a mobility ratio of *µ*_armchair_/*µ*_zigzag_ ≈ 2. Our measured mobility ratio at room temperature is ≈1.85 (Figure 6d), which exhibits exciting accordance to the theoretical calculations. It is interesting that the mobilities along all both directions decrease slightly as temperature reduced from 300 to 60 K (Figure 6c), suggesting that the mobility limited by scattering from charged impurities becomes the dominating scattering mechanism.^59^ Furthermore, the mobility ratio of *µ*_armchair_/*µ*_zigzag_ increases from 1.85 to 3.15 with temperature ranging from 300 to 60 K, which may result from the different temperature dependence of the effective masses along different directions.^60^

## 3 Conclusions

In summary, we have fabricated a 2D GeSe based phototransistor with ultrahigh responsivity of ≈1.6 × 10^5^ A W^−1^. This high responsivity may result from highly efficient light absorption and enhanced photoconductive gain due to the existence of trap states. The exfoliated GeSe nanosheet was found to be cleaving along the [001] (armchair direction) and [010] (zigzag direction) confirmed by TEM and anisotropic Raman characterizations. This GeSe‐based phototransistor also shows strong polarization‐dependent photoresponse with a peak/valley ratio of 1.3. Furthermore, the mobility along armchair direction was measured to be 1.85 times larger than that along the zigzag direction. This work renders 2D GeSe with ultrahigh responsivity as a promising building block for optoelectronics.

## 4 Experimental Section

*Device Fabrication*: Multilayer GeSe was exfoliated from the commercial bulk crystals (2D semiconductors) and transferred to a silicon substrate (300 nm SiO_2_ on the surface) assisted by scotch tape. Then the electrode patterns were fabricated by an electron‐beam lithography system (FEI Quanta 650 SEM and Raith Elphy Plus) and Cr/Au (10/50 nm) metals were deposited by the thermal evaporation (Nexdap, Angstrom Engineering). In order to remove the residual contamination and enhance the metal–semiconductor contact, the fabricated device was subsequently annealed at 300 °C for 1 h in a gas flow of Ar/H_2_ (100/5 sccm).

*Device Characterizations and Measurements*: The morphology and thickness were determined by an optical microscope (BX51, Olympus) and atomic force microscope (Dimension Icon, Bruker). Temperature and polarization‐dependent Raman spectra were performed by a confocal Raman system (Alpha 300R, WITec) with 532 nm laser source. The electrical tests were realized by a semiconductor system (B1500A, Keysight), and the device is in a probe station (CRX‐6.5 K, Lake Shore). For photodetection test, a fixed wavelength of 532 nm laser with tunable power intensity was used as light source.

## Conflict of Interest

The authors declare no conflict of interest.

## Supporting information

Supplementary
