# Supplementary material for: Highly Anisotropic GeSe Nanosheets for Phototransistors with Ultrahigh Photoresponsivity
Source: Adv Sci (Weinh). 2018 Jun 21;5(8):1800478. doi: 10.1002/advs.201800478 (PMC6096999; doi:10.1002/advs.201800478)
Supplement: Supplementary file 1 — Supplementary [file ADVS-5-1800478-s001.pdf]

Copyright WILEY-VCH Verlag GmbH & Co. KGaA, 69469 Weinheim, Germany, 2018.

ADVANCED SCIENCE
Open Access

Supporting Information

for *Adv. Sci.,* DOI: 10.1002/advs.201800478

Highly Anisotropic GeSe Nanosheets for Phototransistors with Ultrahigh Photoresponsivity

*Xing Zhou, Xiaozong Hu, Bao Jin, Jing Yu, Kailang Liu, Huiqiao Li, and Tianyou Zhai**

Copyright WILEY-VCH Verlag GmbH & Co. KGaA, 69469 Weinheim, Germany, 2013.

Supporting Information

Highly Anisotropic GeSe Nanosheet for Phototransistor with Ultrahigh Photoresponsivity

*Xing Zhou, Xiaozong Hu, Bao Jin, Jing Yu, Kailang Liu, Huiqiao Li, and Tianyou Zhai**

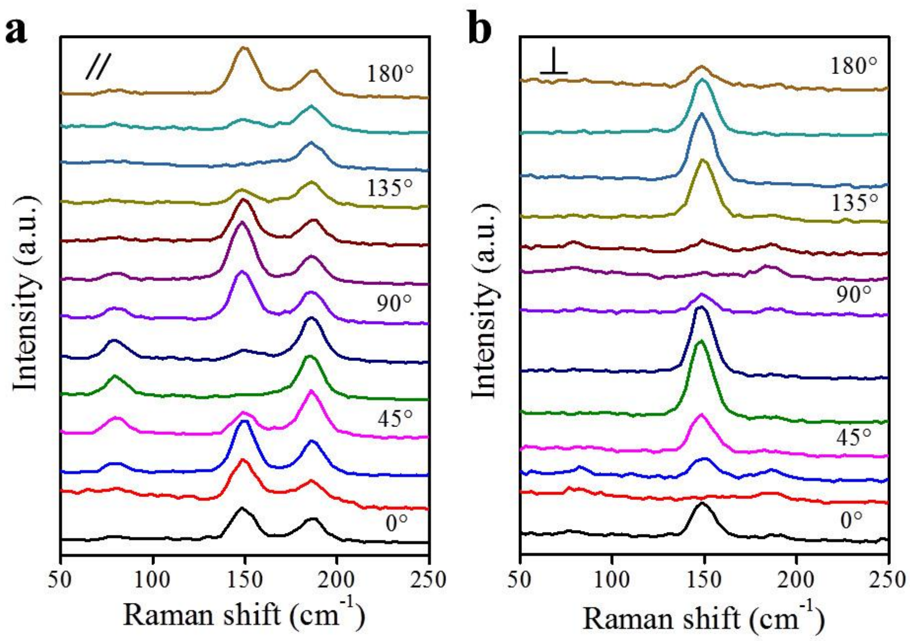

Figure s1. a, b) Angle-dependent Raman spectra under prallel and cross configurations, respectively.

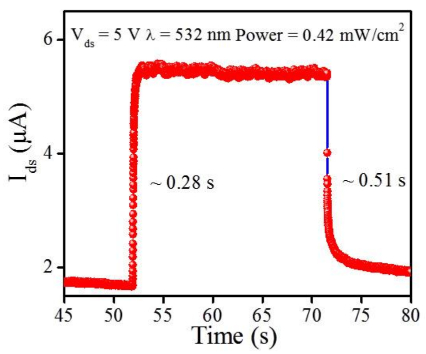

Figure s2. One cycle of time-resolved photoreponse.

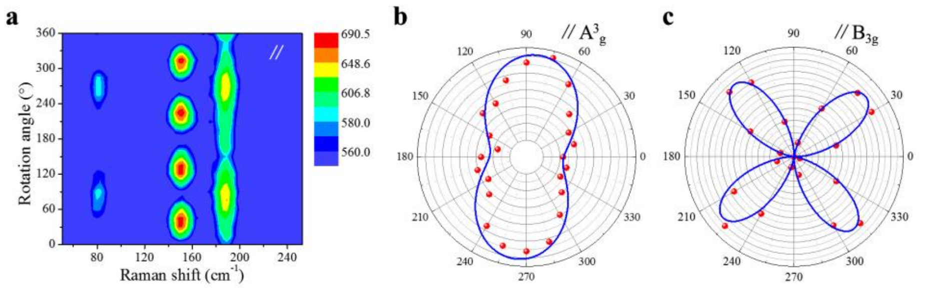

Figure s3. a) False-color plot of the polarized Raman spectra under parallel configuration. b) Angle-dependent Raman scattering intensity of A^3_g and B_{3g} modes.

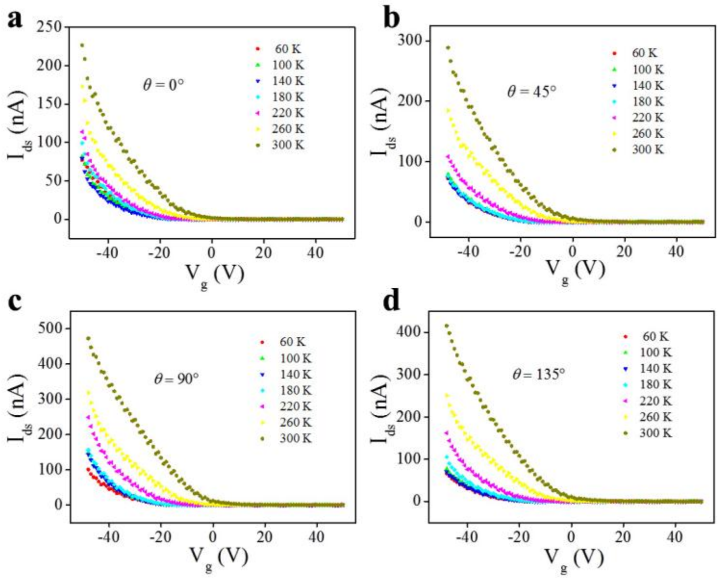

Figure s4. Angle-dependent transfer characteristics along different directions. a-d) θ =0°, 45°, 90°, 135°.
